# Mesenchymal stem cells derived extracellular vesicles improve behavioral and biochemical deficits in a phencyclidine model of schizophrenia

**DOI:** 10.1038/s41398-020-00988-y

**Published:** 2020-09-01

**Authors:** Hadas Tsivion-Visbord, Nisim Perets, Tamar Sofer, Lior Bikovski, Yona Goldshmit, Aangela Ruban, Offen Daniel

**Affiliations:** 1 Sackler Faculty of Medicine, Tel Aviv University, Tel Aviv, Israel; 2 Sagol School of Neuroscience, Tel Aviv University, Tel Aviv, Israel; 3 Departments of Medicine and of Biostatistics, Harvard University, Boston, MA USA; 4 The Myers Neuro‐Behavioral Core Facility, Sackler School of Medicine, Tel Aviv University, Tel Aviv, Israel; 5 School of Behavioral Sciences, Netanya Academic College, 4223587 Netanya, Israel

**Keywords:** Stem cells, Molecular neuroscience

## Abstract

Schizophrenia is a debilitating psychiatric disorder with a significant number of patients not adequately responding to treatment. Phencyclidine (PCP) is used as a validated model for schizophrenia, shown to reliably induce positive, negative and cognitive-like behaviors in rodents. It was previously shown in our lab that behavioral phenotypes of PCP-treated mice can be alleviated after intracranial transplantation of mesenchymal stem cells (MSC). Here, we assessed the feasibility of intranasal delivery of MSCs-derived-extracellular vesicles (EVs) to alleviate schizophrenia-like behaviors in a PCP model of schizophrenia. As MSCs-derived EVs were already shown to concentrate at the site of lesion in the brain, we determined that in PCP induced injury the EVs migrate to the prefrontal cortex (PFC) of treated mice, a most involved area of the brain in schizophrenia. We show that intranasal delivery of MSC-EVs improve social interaction and disruption in prepulse inhibition (PPI) seen in PCP-treated mice. In addition, immunohistochemical studies demonstrate that the EVs preserve the number of parvalbumin-positive GABAergic interneurons in the PFC of treated mice. Finally, MSCs-EVs reduced glutamate levels in the CSF of PCP-treated mice, which might explain the reduction of toxicity. In conclusion, we show that MSCs-EVs improve the core schizophrenia-like behavior and biochemical markers of schizophrenia and might be used as a novel treatment for this incurable disorder.

## Introduction

Schizophrenia is a severe psychiatric disorder, ranked by the World Health Organization as one of the top ten illnesses contributing to the global health burden of disease^1^. Major areas of disability are largely divided into positive, negative and cognitive symptoms. Negative symptoms underlie anhedonia, impaired vocational and social functioning and are a strong predictor of poor functional outcome^2,3^. Antipsychotic medications are currently the most commonly used from first episode diagnosis^4^. Unfortunately, roughly a third of patients do not respond well to antipsychotics and are considered treatment-resistant^5,6^, while responders frequently suffer adverse effects, such as weight gain and dyslipidemia, which may result in severe deterioration of health after long-term treatment^7^. This underlines the fact that adequate therapies are still sorely lacking for this disorder.

A possible and innovative potential therapy is the use of Human Mesenchymal stem cells (MSCs) or their derivatives. MSCs-based therapy has achieved positive effects in various animal models of disease and in several human clinical trials^8^. MSCs are a population of undifferentiated adult stem cells that can self-renew and differentiate into multiple lineages^9–12^. MSCs are simple to obtain and manage, have low immunogenicity and are able to cross the blood brain barrier. These features make them a promising approach for treatment, especially for CNS and neurodegenerative disorders, for which stem cell therapy has been largely pursued.

Indeed, MSCs have been shown to have beneficial effects in a large number of disease models, and specifically for CNS disorders^9–11,13^. MSCs were also previously utilized in our lab as experimental treatment for mice, in models of psychiatric disorders. The MSCs were transplanted to the cortices of model mice and shown to protect against phencyclidine (PCP)-made deficits in a model of schizophrenia^14,15^, as well as mitigate autistic related behaviors in a BTBR mouse model of autism^16^. Current evidence indicates that MSCs affect damaged tissue via secretion of paracrine factors and stimulation of host cells^17^. These effects have been suggested to be mediated by EVs (extra-cellular vesicles), secreted by the MSCs^18,19^. EVs are heterogeneous vesicles that are bound by a phospholipid bilayer that function as mediators of inter- cellular communication via their loaded proteins, RNA and/or DNA^20^. Moreover, EVs derived from MSCs have been reported to have therapeutic effect in preclinical studies in diverse tissues and indications, including the treatment of diseases in the CNS^15–27^.

In an experiment recently performed in our lab, MSCs-EVs were administered using an intranasal approach to mice modeling autism in a BTBR model. The study demonstrated relief of autistic-like behaviors^28^, such as repetitive behavior and social interaction. Intranasal delivery was also shown in other preclinical studies to lead to increased brain accumulation of EVs^29^ and provide neuroprotection^30,31^. As of today, there are no clinical trials employing intranasal administration of EVs (http://clinicaltrials.gov), while a few trials use intravenous application (such as NCT02138331 for Diabetes Mellitus Type 1). The intranasal approach of administration provides better access to the CNS for non-invasive, direct delivery to the brain^32^, is likely to be well tolerated by patients and therefore has tremendous translational potential.

Use of phencyclidine (PCP), a N-methyl-D-aspartate (NMDA-R) receptor antagonist, has been validated in rodents as a model for schizophrenia, shown to reliably induce positive, negative and cognitive-like behaviors in a multitude of behavioral tests^33–35^. Sub-chronic administration of PCP in rodents was shown to cause reduced social interaction^36^, hypofrontality^37^, and to reduce parvalbumin-positive interneurons in the prefrontal cortex (PFC)^38^. Here we used PCP to induce schizophrenia-like behaviors in mice and utilized intranasal administration of MSCs-derived EVs as a therapeutic tool delivered to their brains. Auspiciously, the EVs ameliorated behavioral and biochemical deficits in our preventative model. This proof-of-concept study may indicate a novel future application for MSC-EVs in the treatment of schizophrenia and other CNS disorders.

## Materials and methods

### Mice

C57Bl/6J mice (Envigo, Jerusalem, Israel) aged 7 weeks were placed under a 12-h light/12-h dark condition in a temperature and humidity-controlled facility with food and water ad libitum. Mice were reared in social groups of 4–5 mice per cage. All experimental manipulations were performed in accordance with guidelines and regulations of the Tel Aviv University Committee of Animal Use for Research and Education.

### Experimental design

**Fig. 1:**
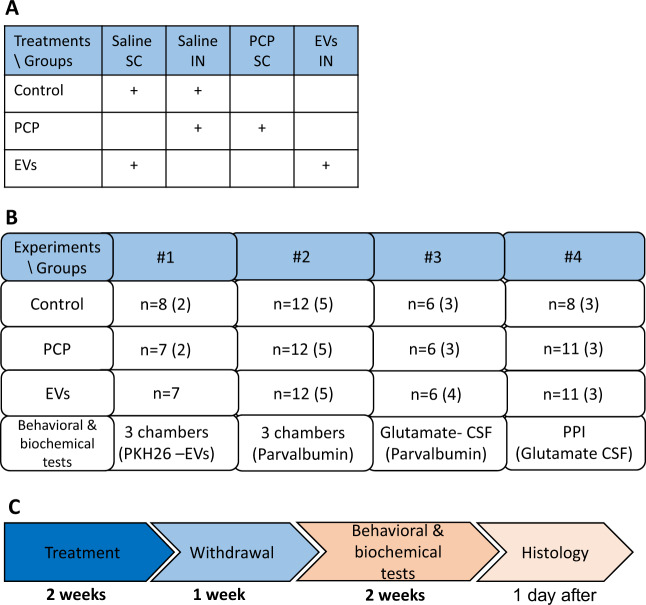
Experimental design. **a** Table providing the treatment conditions compared in the study. Control conditions included subcutaneous (SC) and intranasal (IN) administrations of saline, the PCP condition included SC injections of PCP and IN administration of saline, and the EVs condition included SC injections of PCP and IN administration of MSCs-EVs. **b** the number of mice in each of four experiment replications, by condition, and the behavioral and biochemical tests they were subject to. In each experiment, all mice participated in one test, and some mice (in parenthesis) further participated in another test. For histology assessments the mice were sacrificed one day following behavioral tests (Ex. 1–3 in parenthesis). **c** Timeline of each experiment. 7-weeks-old mice were allocated at random to three treatment conditions described in **a**. Treatment conditions were applied once daily for 14 days, followed by a withdrawal period of 7 days. Behavioral and biochemical tests were performed within 2 weeks following the withdrawal period.

The experiment was performed in four replications with a total of 106 male C57BL/6 mice (Fig. 1). At 7-weeks-old the mice were allocated at random for treatment groups, defined according to three experimental conditions described henceforth. EVs treated group: PCP (Sigma-Aldrich, St. Louis, MO, USA) at a dose of 10 mg/ kg dissolved in 0.9% normal saline was injected subcutaneously once daily for 14 days. On each of the 14 days of PCP administration, EVs derived from MSCs were administered intranasally (2.4 × 10^7^ EVs/mouse/day). PCP-treated group: Mice were treated with PCP in the same manner as the EVs-treated group and administered with equal volume of saline intranasally. Control group: Mice were injected subcutaneously and treated intranasally with saline only. One week after the last dose of PCP, mice underwent behavioral tests. Sample sizes for the behavioral experiments were chosen based on previous studies. One day after completion of behavioral testing the mice were sacrificed and mice from each group were selected randomly for histology assessments.

One animal from the PCP-treated group was sacrificed due to irregular behavior during the model-induction phase. No other animals died during the experiment. The investigators were not blind to the group allocation, but the data acquisition was completely automatic.

### Behavioral testing

#### 3 chambers social interaction

Impaired social behavior is one of the most defining characteristics of schizophrenia^39^ and PCP has been found to reliably induce social interaction deficits in rodents^40^. We evaluated the sociability of the treated mice groups by performing the three-chamber test, as reported elsewhere^15,41^. The apparatus consisted of an opaque white Plexiglas arena divided into one central and two lateral compartments. The lateral compartments each contained a single plastic cup, and the lateral walls of the central compartment had openings for the mice to pass through. Mice were habituated for 20 min/day for 2 consecutive days prior to the test day, when they were free to explore the middle chamber. On the test day, the test mouse was initially habituated to the arena for 10 min, during which time two black partitions completely covered the sides of the arena containing the cups. Thereafter, the Plexiglas partitions were removed, and an unfamiliar naive male C57BL/6 mouse was placed in one cup (social stimulus). The other cup remained empty (inanimate stimulus). The mice were free to explore all chambers for 10 min, during which time they were videotaped. The position of the object mouse was altered between left and right chambers between subjects testing. Video files were analyzed with Ethovision 13 software (Noldus, Wageningen, The Netherlands). To calculate the results, we used the preference index (S − NS)/(S + NS), where S is the time spent in the social chamber and NS is the time spent in the non-social chamber.

#### Prepulse inhibition of the acoustic startle

Sensorimotor gating was assessed using the paradigm of prepulse inhibition (PPI) of the acoustic startle response, in which a weak prepulse stimulus suppresses the response to a subsequent startling pulse stimulus. Prepulse inhibition of the acoustic startle reflex is believed to have face, construct, and predictive validity for modeling schizophrenia^42^. In healthy humans as well as rodents, the prepulse stimulus serves to attenuate the reflexive response to the pulse^43^ and deficits are observed in several psychiatric populations, most notably in patients with schizophrenia^42,44^.

PPI test was performed as previously described^45^. Startle chamber (San Diego Instruments) was used for measuring startle reactivity. The startle chamber comprised a nonrestrictive cylindrical enclosure made of clear Plexiglas attached horizontally on a mobile platform, which in turn was resting on a solid base inside a sound-attenuated isolation cubicle. A high-frequency loudspeaker mounted directly above the animal enclosure inside each cubicle produced a continuous background noise of 65 dB (A-scale) and the various acoustic stimuli in the form of white noise.

Subjects were presented with a series of discrete trials comprising a mixture of four types of trials. These included pulse-alone trials, prepulse-plus-pulse trials, prepulse-alone trials, and trials in which no discrete stimulus, other than the constant background noise, was presented (denoted here as “no-stimulus” trials). A reduction of startle magnitude in prepulse-plus-pulse trials relative to those in pulse-alone trials constitutes PPI. The pulse stimulus employed was 120 dB in intensity and 40 ms in duration. Prepulses of various intensities were employed: 69, 73, 77, 81, and 85 dB, which corresponded to 4, 8, 12, 16, and 20 dB above background, respectively. The duration of prepulse stimuli was 20 ms. The stimulus onset asynchrony of the prepulse and pulse stimuli on prepulse-plus-pulse trials was 100 ms.

A session began with the animals being placed into the Plexiglas enclosure. They were acclimatized to the apparatus for 2 min before the first trial began. The first six trials consisted of startle-alone trials, which served to habituate and stabilize the animals' startle response. Subsequently, the animals were presented with 12 blocks of discrete test trials. Each block consisted of one trial of each of the following trial types: pulse-alone, prepulse-plus-pulse trials of each of the five levels of prepulse, prepulse-alone of each of the five levels of prepulse, and no stimulus (i.e., background alone). The session was concluded with the final block of six consecutive startle-alone trials. The interval between successive trials was variable with a mean of 15 s (ranging from 10 to 20 s).

% PPI = ((mean startle response to 120 dB pulse alone-mean startle response following a prepulse)/mean startle response to 120 dB pulse alone) *100.

#### Isolation and identification of extracellular vesicles

Human MSCs were purchased from Lonza (Basel, Switzerland) and have tested negative for mycoplasma, bacteria, yeast, and fungi. In addition, HIV-1, hepatitis B and hepatitis C are not detected for all donors and/or cell lots. Cells were cultured and expanded as previously described^46^. Prior to EVs collection, the cells were cultured in EVs-free medium, and 3 days later, the medium was collected.

The EVs were purified by isolating the culture fluid and centrifuging for 10 min at 300 *g*. The supernatant was recovered and centrifuged for 10 min at 2000 *g*. Once again, the supernatant was recovered and centrifuged for 30 min at 10,000 *g*. The supernatant was taken, put through a 0.22 µm filter, and centrifuged for 70 min at 100,000 *g*. The pellet, containing the EVs and proteins, was washed in PBS then centrifuged for 70 min at 100,000 *g*. The pellet, containing the purified EVs, was resuspended in 200 µl of sterilized PBS. Each centrifugation was conducted at 4 °C.

The size and concentration of MSCs-EVs were determined with use of the Nanosight (Merkel Technologies Ltd., Israel). FACS analysis was performed to verify the specific EVs surface markers, including CD63 and CD81 (Supplementary fig. S1).

#### Flow cytometry

For flow cytometry analysis, EVs were coated onto 4-μm-diameter aldehyde/sulfate latex beads. 50 µl EVs were incubated with 12.5 µl 4-μm-diameter aldehyde/sulfate latex beads (cat# A37304, Invitrogen) for 15 min at room temperature. 700 µl sterile PBS was added, and the mixture was then transferred to 4 °C and gentle shaking overnight. After centrifugation, the pellet was blocked by incubation with 200 µl 100 mM glycine for 30 min at room temperature. EVs-coated beads were washed in PBS and resuspended in 100 µl sterile PBS. Afterwards, beads were incubated with CD63-APC (cat#130-118-078, Miltenyi biotec), CD81-APC (cat# 130-119-787 Miltenyi biotec) or IgG1 Isotype control (cat#130-113-434, Miltenyi biotec) fluorescent Abs for 15 min on ice in the dark. Beads were analyzed by flow cytometry using Gallios flow analyzer FACS (Beckman Coulter). Data were analyzed using the Kaluza Analysis Software (Beckman Coulter).

#### EVs labeling

EVs were labeled with PKH26 (Sigma-Aldrich)^47,48^. PKH26 (2 µl) in 500 µl diluent was then added to 50 µl EVs in PBS for 5 min of incubation. EVs were suspended in 70 ml PBS and were centrifuged for 90 min at 100,000 *g* at 4 °C. The pellet was suspended in 200 µl of PBS.

#### CSF sampling and HPLC analysis of glutamate in CSF

We sampled CSF in order to evaluate the impact of our treatment on CNS Glutamate levels. We sampled the CSF one day after completion of the last behavioral test, whereby the mice were also sacrificed. Briefly, after the mice were anesthetized with a mixture of ketamine/xylazine (ketamine 100 mg/kg, xylazine 10 mg/kg), CSF was immediately collected from the cisterna magna using a glass capillary tube^49^. Typically, 5–15 ml of CSF was collected, immediately frozen in liquid nitrogen and stored at −80 °C. Blood-contaminated samples were not analyzed. CNS Glu level was analyzed with pre-column derivatization with OPA reagent and separated by reversed-phase HPLC with a scanning fluorescence detector. The excitation and emission wavelengths were 350 and 460 nm, respectively. Chromatography was performed using the UltiMate 3000 LC system (Thermo Scientific). The amino acid standard mix (Sigma-Aldrich) or samples were precolumn derivatized with OPA reagent solution. The derivatization reagent was 5.0 mg OPA dissolved in 100 µL of methanol, and diluted with 900 µL of 0.4 M borate buffer (pH 9.5) and 5 µl of b-mercaptoethanol, freshly prepared every 48 h and protected from light exposure. Derivatization of amino acids: A standard or sample was mixed with 5 µl of OPA derivatization reagent in the autosampler. The mixture was vortexed for 2 min before HPLC analysis. All chromatographic separations were performed on a Thermo Hypersyl Gold 5U column (4.6 mm × 250 mm, 2.5 µl). The amino acid concentration was determined using the peak area and the external standard method.

##### Immunohistochemistry

At the endpoint of the behavioral experiments, animals were transcardially perfused, under ketamine/xylazine anesthesia, with cold phosphate-buffered saline (PBS) followed by 4% paraformaldehyde in phosphate buffer. The brains were immersed in 4% paraformaldehyde for 24 h at 4 °C followed by cryoprotection in 30% sucrose for an additional 48 h. For whole-brain imaging and EVs staining, the mice (*N* = 2 each for control and PCP groups) were given 5 µl of labeled EVs via intranasal administration and perfused 24 h later. Whole brain fluorescence imaging was taken with Maestro CRi, excitation filter 523, and emission filter 560. For immunostaining analysis, the brains were frozen in chilled 2-methylbutane (Sigma-Aldrich) and subsequently sectioned into sagittal slices measuring 10 µm, using a microtome cryostat.

Due to the well-known decrease in parvalbumin (PV) expression in the PFC of schizophrenic patients^50^, as well as with the use of NMDA receptor antagonists^51^, the number of PV positive cells were quantified. For PV staining, brains were embedded in OCT, and 20 μm slices were collected for free-floating immunohistochemical staining. Sections were washed in 1XPBS and blocked with 2% normal goat serum (Sigma-Aldrich) containing 0.3% TritonX-100 for 1 h at room temperature. Primary antibody (anti-PV 1:1000 (Abcam, Cambridge, MA, USA; ab11427) was diluted in the blocking solution and incubated for overnight at 4 °C followed by incubation with AlexaFluor 488 goat anti-rabbit immunoglobulin for 1 h at room temperature. Slices were counterstained with DAPI (1:500; Sigma-Aldrich) and mounted with fluorescent mounting solution (Fluoromount-G, SouthernBiotech) and covered with a cover slide. Images were obtained using the Axio Imager.Z2 microscope (Zeiss, Thornwood, NY).

All images were analyzed using ImageJ software. The acquired images were converted to TIFF format, and cell counts for PV and DAPI were performed on 20× images using the thresholding function in Image J (NIH). Following assessment of several sections, threshold values of pixel optical densities were defined to obtain clear cell outlines while minimizing identification of non-somatic staining. The same pixel threshold values were then applied to all sections. For the analysis of PCP-induced changes, the percentages of PV + cells among all DAPI-stained cells were calculated. Four sections for each mouse brain were analyzed and a mean value used in the final assessment.

### Statistical analysis

We used one-way analysis of variance (ANOVAs) using a commercial software (GraphPad Prism 6), to assess differences between the three treatment groups, followed by Fisher’s LSD post hoc test.

For the PPI test, to consider the experimental design with two factors (treatment and prepulse level), unequal sample sizes in the treatment factor, and same mouse exposed to multiple prepulse levels (repeated measures), we used linear mixed models with random mouse effects, followed by ANOVA and post-hoc analysis (*F* test) of difference in response between treatment groups within noise levels. The analysis was performed using the R package lme4.

All data are represented as mean ± SEM, with *p* < 0.05 being considered statistically significant. There were no deviations from normality, according to the Shapiro–Wilk test.

## Results

### MSC-EVs-treated mice show amelioration in impaired schizophrenic behavioral phenotype

**Fig. 2:**
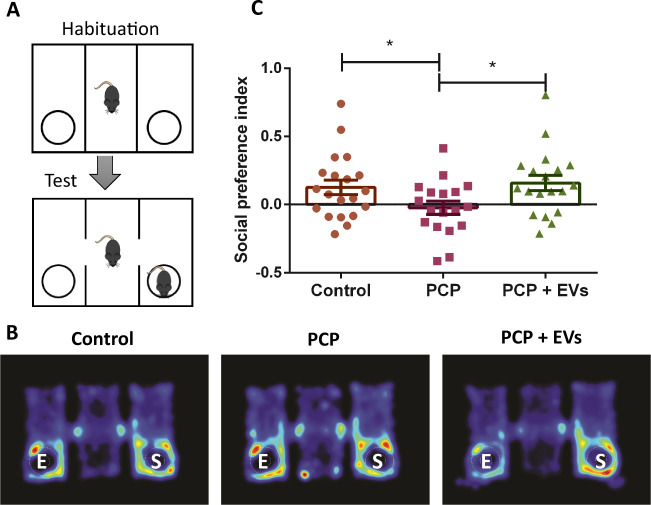
Three chamber social approach test. **a** Mice were habituated for 10 min to the middle chamber, followed by 10 min in their social preference was indexed. PCP-treated mice display deficits in social behavior shown as decreased amount of time spent making contact with a stranger mouse as compared with controls. MSCs-EVs treatment significantly restored a normal social pattern in these mice, as shown in a heat map representation of the time spent exploring the chambers (**b**) and by quantitative measurement of that activity (**c**). Time spend in close proximity to the social cage and the nonsocial cage were measured with preference index; (S − NS)/(S + NS), where S is the time spent in the vicinity of the social stimulus and NS, the time spent in the vicinity of the non-social stimulus. Results displayed as mean ± SEM. Statistical analysis: One-way ANOVA with a post hoc Fisher’s LSD test. *p < 0.05.

We evaluated the sociability of the treated mice groups in the three-chamber test. We detected no difference in total locomotor activity among the groups in the first 10 min, when the mice were limited to the central zone with no social/nonsocial stimulus (Supplementary fig. S2). Thereafter, when the mice were allowed to explore both the chamber with the social stimulus and the chamber with the nonsocial stimulus, a one-way ANOVA revealed a significant effect of treatment (*F* = 3.311, DF = 2.54, *p* < 0.05). Post-hoc analysis demonstrated that PCP-treated mice did not show a preference for the social stimulus, while the control mice and the EVs-treated mice both showed a clear preference for the social stimulus cage (*p* < 0.05, Fig. 2).

**Fig. 3:**
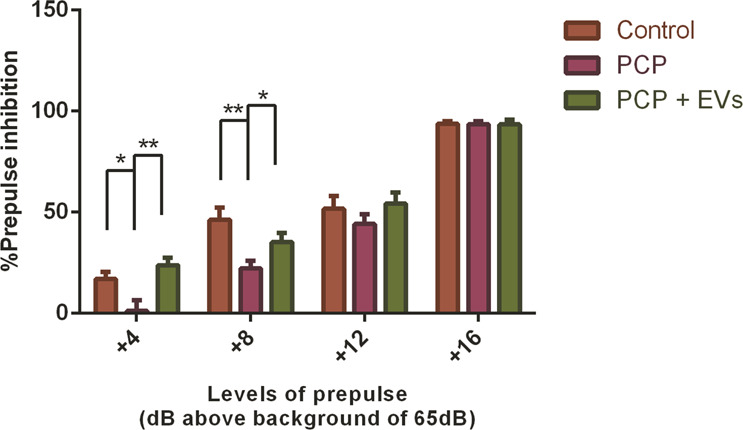
Prepulse inhibition test. PPI data (mean ± SEM) show the percent of prepulse inhibition of the startle response following the presentation of prepulse-plus-pulse acoustic stimuli. Four different prepulse intensities (69, 73, 77, and 81 dB) were measured. PCP-treated mice exhibited deficits in sensorimotor gating as measured through the PPI test, for two of the prepulses tested (69 and 73 dB). MSCs-EVs treatment significantly restored the percentage of PPI for these prepulses. Statistical analysis: linear mixed models with random mouse effects, followed by ANOVA and post-hoc analysis. Results displayed as mean ± SEM. *p < 0.05, **p < 0.01.

As for the PPI test, in the pulse-alone trials, there was no difference between the reactivity to the startle of the different treatment groups (*F* = 0.4726, DF = 2.26, *p* = 0.6286). In addition, a 3 × 4 ANOVA revealed a highly significant effect of the of the intensity of the prepulse (*F* = 201.49, DF = 3.78, *p* < 0.001), reflecting the fact that the percent inhibition increased as a function of the prepulse intensity; this is considered as a substantial validation of the procedure. Most importantly, we saw a significant effect of the interaction between treatment and prepulse (*F* = 2.5064, DF = 6.78, *p* = 0.02). Consequently, post hoc separate analyses were carried out for each of the prepulse intensities and it was demonstrated that at prepulses 69 and 73 there was a significant reduction in %PPI in the PCP-treated group, while the EVs treatment ameliorated significantly this disruption (Fig. 3).

### MSC-EVs improve the reduction in parvalbumin (PV)-positive interneurons in the PFC of PCP-treated mice

**Fig. 4:**
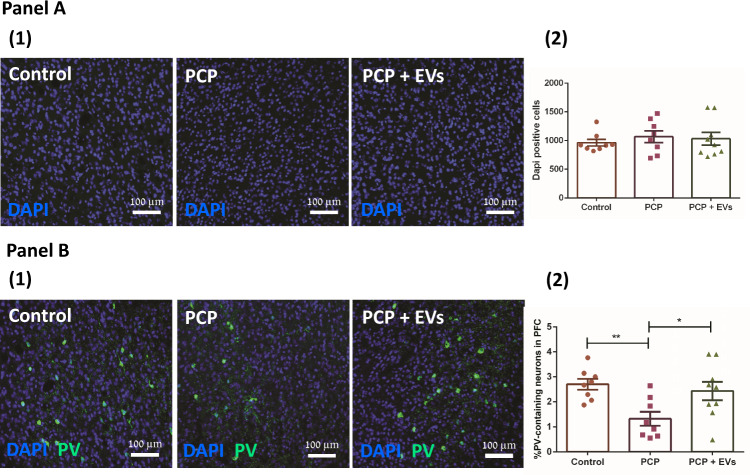
Effects of PCP treatment on PV-positive interneurons in the PFC. **a** Representative sample images (1) and group data summary (2) of Dapi-positive cells from control, PCP and EVs-treated mice in the PFC, showing no significant difference in nuclei number between groups. **b** Representative sample images (1) and group data summary (2) of number of parvalbumin (PV)-positive cells in control, PCP and EVs-treated groups, showing a reduced number of PV + interneurons in the PCP treated group, while EVs treated group did not differ from control. Results displayed as mean ± SEM. Statistical analysis: One-way ANOVA with a post hoc Fisher’s LSD test. *p < 0.05.

To elucidate the cellular alterations that may be associated with the behavioral changes following the EVs treatment, we performed a histological study (Fig. 4). First, we quantified the number of Dapi-positive cells in the PFC (Fig. 4a) and found no significant difference between the experimental mice groups (*F* = 0.3034, DF = 2.22, *p* = 0.7414). We then quantified the number of PV-positive cells and found a significant effect of treatment (*F* = 5.621, DF = 2.22, *p* = 0.01). Following with post-hoc analysis, we found a marked decrease in PV-positive interneurons in the PFC of PCP-treated mice (*p* < 0.01). However, in the EVs treated mice we observed preservation in the number of PV-positive staining, indicating the EVs protect against damage to the GABAergic cells (Fig. 4b).

### MSC-EVs alleviate the reduction in glutamate concentration the CSF of PCP-treated mice

**Fig. 5:**
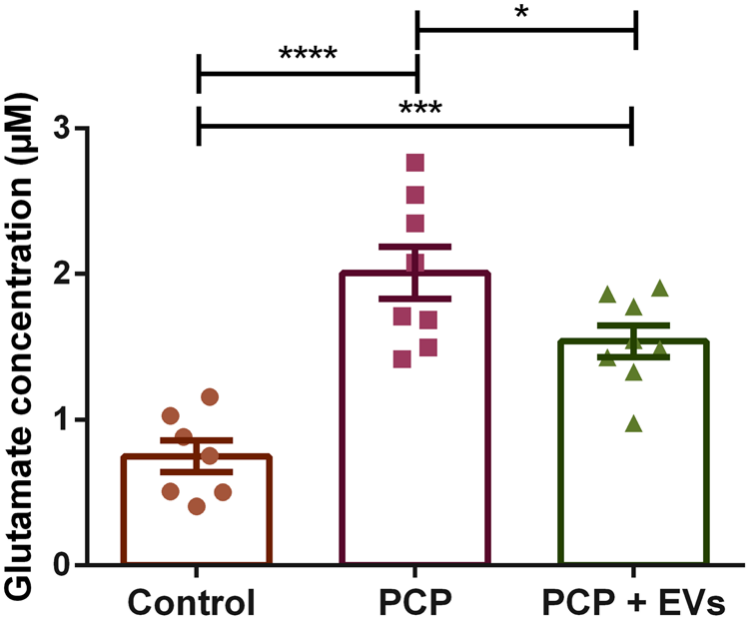
Glutamate levels in the CSF. PCP-treated mice displayed a very significant increase in glutamate levels in the CSF, while MSCs-EVs treatment significantly restored glutamate levels. Results displayed as mean ± SEM. Statistical analysis: One-way ANOV with a post hoc Fisher’s LSD test. *p < 0.05, ***p < 0.001, ****p < 0.0001.

To further study the possible association between the behavioral improvement and the biochemical alterations in the PCP-treated mice, we examined glutamate levels in the CSF of the mice (Fig. 5).

One-way ANOVA shows a highly significant effect of treatment group (*F* = 20.27, DF = 2.20, *p* < 0.0001). Post-hoc analyses showed an increase in glutamate levels in the CSF of PCP-treated mice (*p* < 0.0001) and EVs-treated mice (*p* < 0.001). Most importantly, the post-hoc showed a difference between the PCP and EVs-treated groups (*p* = 0.024), suggesting that the EVs has a mitigating effect.

## Discussion

In the current study we have shown that intranasal treatment with EVs derived from MSCs can improve schizophrenia-like behaviors and biochemical properties in a PCP mice model. The EVs ameliorated deficits in social interaction and PPI, prevented reduction in Parvalbumin-expression GABAergic interneurons in the PFC and also had a mitigating effect on glutamate levels in the CSF of PCP treated-mice. Previous studies performed in our lab^18,19^ have shown the benefit of using MSCs by intracranial transplantation in a PCP mice model. In this study we demonstrate that, easily delivered by an intranasal approach, MSCs-EVs constitute a marked improvement for therapy in a schizophrenia model.

The intranasal route for administration of drugs is especially advantageous for CNS access, convenient to use and therefore can improve patient compliance. Another advantage of using MSCs-EVs is their ability to migrate to the site of injury, demonstrated in studies done by our research group and by others. In a study designed to determine the specific migration of EVs in different mice models of pathology, the EVs were shown to home on relevant areas, such as migration to the area of injection in an endothelin-1 model of stroke, and the hippocampus in a transgenic 5XFAD model of Alzheimer’s^52^. In another study, specific accumulation of EVs was detected in kidneys of mice with acute kidney injury (AKI) 24 h after i.v. injection, while no signal was detectable at that time in the healthy mice^53^. Another study showed that significantly more MSCs-EVs were incorporated into neurons in brain areas that are susceptible to neurodegeneration, compared to neurons elsewhere, following insults such as status epilepticus in rats^54^. It is important to note that previous published studies performed in our lab, have observed that when MSCs-derived EVs are administered intranasally to C57BL mice, they do not cause alterations in measured behaviors^28^ and do not show region specific accumulation in the brain over a 24 h period^55^.

We assumed that the PCP-induced injury will not be uniform across the brain, but rather have specific locations. Indeed, we observed, in our model of schizophrenia, we observed accumulation of EVs in the prefrontal cortex of the model mice (Supplementary Fig. S3). EVs accumulation in the PFC might suggest this area to be most sensitive to PCP-induced injury. This finding correlates with other studies pointing to the PFC as a crucial region involved in schizophrenia^56,57^, and PCP-induced psychosis^56,58^. Indications for PFC involvement in schizophrenia are evident in multiple forms. In fMRI studies, patients exhibit abnormal activation of the PFC under demands for working memory^59^. Postmortem studies consistently show a reduction in GAD67 in the PFC^60,61^, and a decrease in PSD95 was shown in mRNA levels in the PFC^62^.

In order to examine the PCP models’ relevance to schizophrenia, we employed several behavioral tests. The three chambers social interaction test, which MSCs treatment has previously shown to improve and the PPI of the acoustic startle reflex, considered as an endophenotype for schizophrenia. Indeed, the PCP-treated mice have shown major deficits in social interaction as well as in PPI, and importantly, the EVs have shown the ability to normalize both behaviors. Since the level of disturbance in social interaction is often used as a measure of negative-like behaviors in animal models of schizophrenia, the improvement seen by the EVs treatment is very promising. In addition, PPI is considered as a core behavioral deficit of schizophrenia. It reflects a complex phenotype that involves diverse neural systems as well as different neurochemical substrates including dopamine, glutamate and GABA^42,63^. Therefore, the amelioration of the deficit in PPI might be explained by the EVs ability to affect several pathways in parallel and asserts their ability to treat this multi-factorial disorder.

In the model we employed, the NMDA receptor antagonist PCP leads to NMDAR hypofunction. NMDA receptor antagonists such as PCP lead to NMDAR hypofunction. Inhibitory GABAergic interneurons which are normally activated by stimulation of NMDAR located on their surface, are subsequently disinhibited by PCP and result in excessive depolarization-dependent release of glutamate and a potential cascade of excitotoxic events, leading to neurotoxic injury^64–66^. This process may be reflected by the levels of glutamate in the CSF of PCP-treated mice in our study.

Hypofunction of NMDA receptors following administration of NMDA receptor antagonists such as PCP, is well known to cause schizophrenia-like behaviors in otherwise healthy subjects. However, the brain areas or cell types responsible for these symptoms’ emergence remain largely unknown. One possibility suggests that hypofunction at GABAergic interneurons plays an important role^67^. This theory is based on various studies which have proposed that since GABAergic interneurons provide inhibitory control of cortical and subcortical circuits^68,69^, they may lead to glutamatergic and dopaminergic dysfunction, which in turn leads to the symptoms of schizophrenia^70,71^. Moreover, a subset of GABAergic interneurons which express parvalbumin (PV), were shown to be consistently decreased in the PFC of schizophrenic patients^72^. Likewise, subchronic PCP treatment was shown to reduce PV levels in rodent PFC^72,73^. GABAergic dysfunction in the PFC was also suggested to be the cause of behavioral deficits induced by prenatal PCP treatment^74^. Considering our finding that the MSCs-EVs migrate to the PFC of PCP-treated mice, we examined the PV-expressing GABAergic neurons in the PFC of mice in our experiment. As anticipated, we found a decrease in this subpopulation of neurons in the PCP-treated mice, compared to the control group. As we expected, the treatment with MSCs-EVs seems to restore PV expression in the PFC.

MSCs-derived EVs possess well studied immunomodulatory, anti-inflammatory, and neuroprotective effects^75^. Their neuroprotective qualities have been repeatedly demonstrated both in vitro and in vivo studies^76^. The EVs were shown to increase neuronal survival as well as stimulation of neural cell regeneration in various rodent models of stroke^77,78^. Considering these effects of the MSCs-EVs, we hypothesize that in our model of PCP, the EVs provided protection against loss of PV-expressing GABAergic interneurons. This protection may have led to decreased excitotoxicity, resulting in the decrease in glutamate levels in the PFC of the EVs-treated mice when compared to the PCP-treated group without EVs, and finally manifested in the improved behavior of the EVs-treated mice.

While our results suggest a novel treatment for psychiatric disorders, they should be viewed in light of some limitations. First, we used a preventive approach in this study, as the EVs-treatment was administered simultaneously with PCP injections. It will therefore be up to future studies to establish the treatment potential of MSCs-derived EVs in psychiatric models. Secondly, while we used an established model of schizophrenia with PCP, other models have recently been made prominent, such as maternal immune activation models^79^. Thus, it may be beneficial to examine the EVs as treatment in other models such as these in future research. Finally, while we offer substantial evidence for the EVs biochemical means of action in this mode, the exact mechanism by which they operate remains unknown and further exploration will be needed before clinical application.

In conclusion, our study demonstrates that MSCs-derived EVs improve schizophrenia-like behaviors in a mice model for schizophrenia, thus adding to the large body of evidence that promises these EVs to be an invaluable tool in treating CNS disorders. We demonstrated a possible treatment both for negative and positive symptoms, while using a convenient, non-invasive treatment, which holds great potential for translation into clinical practice. We believe that while the scientific community is still developing understanding of the EVs and their mechanism of action, there is no doubt about their potential to treat various disorders, including schizophrenia.

## Supplementary information

SUPPLEMENTAL MATERIAL
