# Correction: Mesenchymal stem cells derived extracellular vesicles improve behavioral and biochemical deficits in a phencyclidine model of schizophrenia

**DOI:** 10.1038/s41398-020-01016-9

**Published:** 2020-09-22

**Authors:** Hadas Tsivion-Visbord, Nisim Perets, Tamar Sofer, Lior Bikovski, Yona Goldshmit, Aangela Ruban, Offen Daniel

**Affiliations:** 1 Sackler Faculty of Medicine, Tel Aviv University, Tel Aviv, Israel; 2 Sagol School of Neuroscience, Tel Aviv University, Tel Aviv, Israel; 3 Departments of Medicine and of Biostatistics, Harvard University, Boston, MA USA; 4 The Myers Neuro-Behavioral Core Facility, Sackler School of Medicine, Tel Aviv University, Tel Aviv, Israel; 5 School of Behavioral Sciences, Netanya Academic College, 4223587 Netanya, Israel

**Keywords:** Stem cells, Molecular neuroscience

Correction to: *Translational Psychiatry*

10.1038/s41398-020-00988-y, published online 1 September 2020

In the original Article, Dr. Angela Ruban’s name was misspelled as “Aangela Ruban”. This has been corrected in the PDF, HTML, and XML versions of this Article.

