# Supplementary material for: Mesenchymal stem cells derived extracellular vesicles improve behavioral and biochemical deficits in a phencyclidine model of schizophrenia
Source: Transl Psychiatry. 2020 Sep 1;10:305. doi: 10.1038/s41398-020-00988-y (PMC7463024; doi:10.1038/s41398-020-00988-y)
Supplement: Supplementary file 1 — SUPPLEMENTAL MATERIAL [file 41398_2020_988_MOESM1_ESM.docx]

**Mesenchymal Stem Cells Derived Extracellular Vesicles improve Behavioral and Biochemical Deficits in a phencyclidine model of Schizophrenia**

Tsivion-Visbord H^1^., Perets N^2^., Sofer, T^3^., Bikovski L^4^, Goldshmit Y^1^., Ruban A^1^., Offen D^1,2^.

**Supplementary figures**


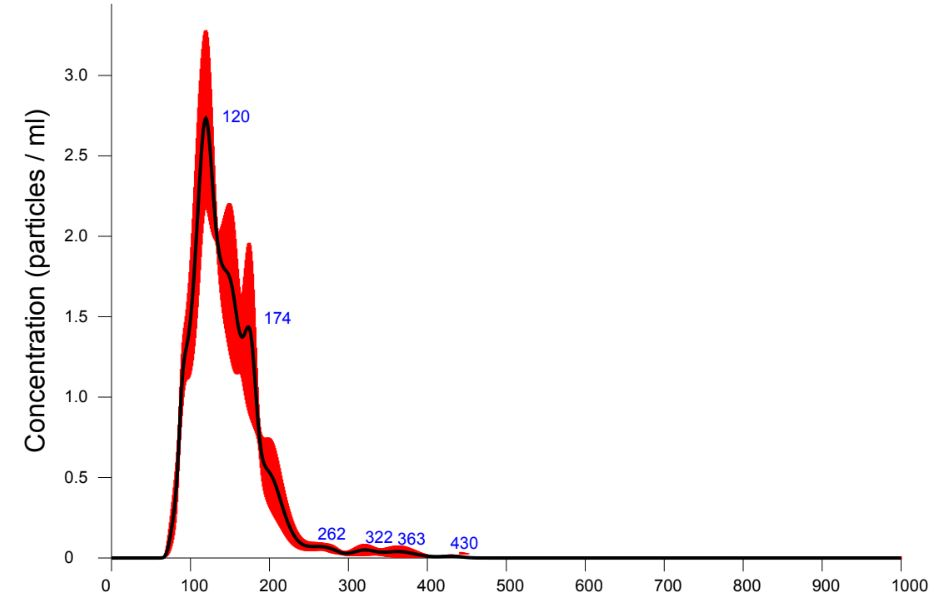

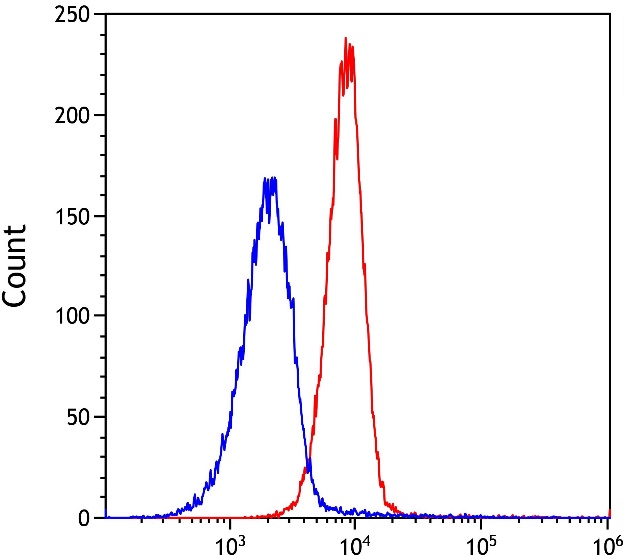

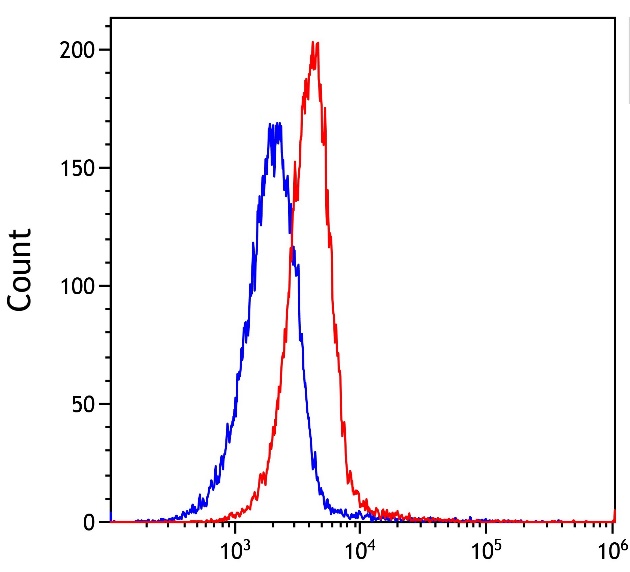
 Supplementary figure 1:

Fluorescence intensity

CD63

CD81

Isotype

Ab

**A**

**B**

Supplementary figure 1: A. Nanosight analysis of MSC-EVs concentration and size distribution. Total concentration was 2.06e+08 +/- 1.39e+07 particles/ml. B. FACS analysis of EVs expression of surface molecules, 50 µl of EVs were incubated with 12.5μl of 4-μm-diameter aldehyde/sulfate latex beads and stained with CD63-APC or CD81-APC Abs (red lines) or negative control IgG1 Isotype Ab (blue line).

Supplementery Figure 2:


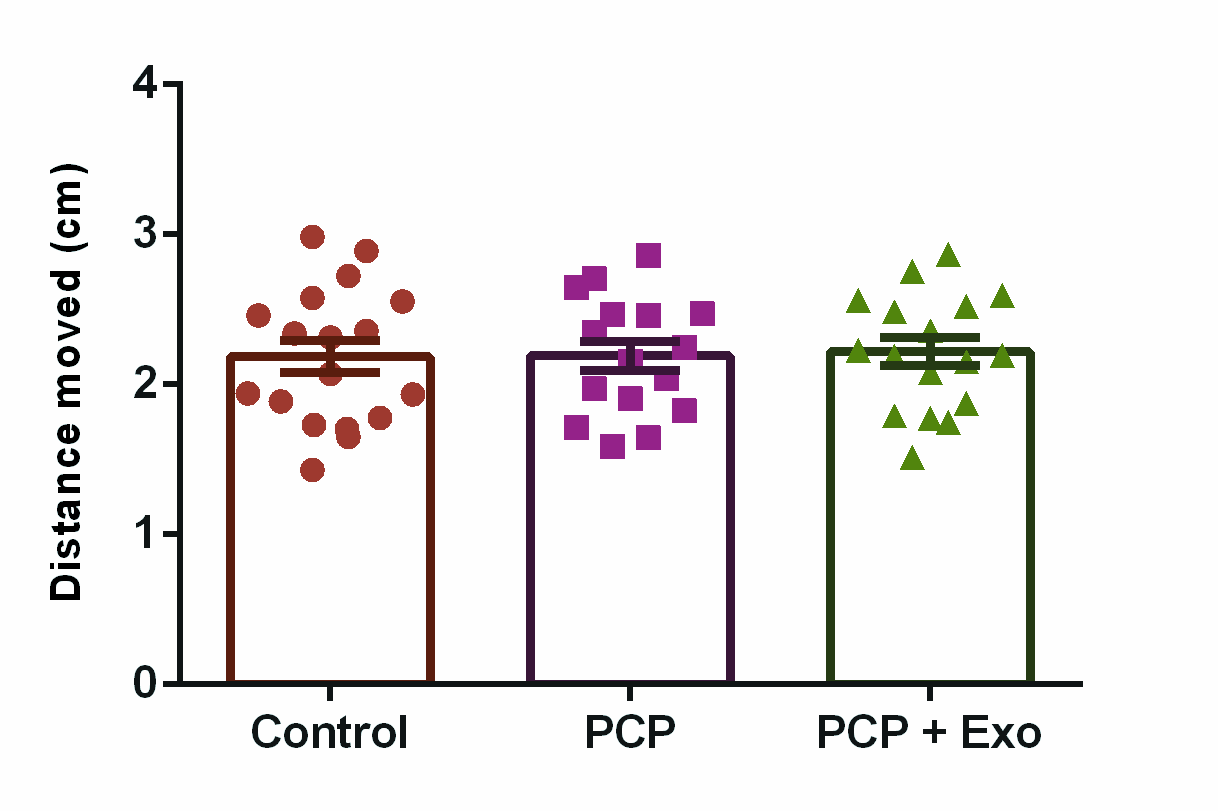


Supplementary figure 2: Three chamber social approach test. Total distance the mice moved during the first 10 min of the social preference test. At this time mice were limited to the central chamber with no social/nonsocial stimulus provided and no difference between the groups was detected. Results displayed as mean ± SEM. Statistical analysis: One-way ANOVA.

Supplementary figure 3: MSCs-derived EVs migration points to the PFC as site of pathology in PCP-treated mice.

MSC-derived EVs were previously shown to migrate to the site of lesion in various models of brain damage44. In this study, we examined whether the MSCs-derived EVs will converge at a specific site at the brains of PCP-treated mice. We used whole brain imaging to visualize PKH26-labeled MSC-EVs 24-h after intranasal administration (Supplementary Figure 2A). Further dissection of the brains and review of different brain regions showed that the MSC-EVs accumulated especially at the PFC of PCP-treated mice, when compared to control mice (Supplementary Figure 2B), pointing to the PFC as the impaired area in the PCP injured brain. The PKH26-labled EVs migrated to the PFC in PCP-treated mice but not to the striatum, hippocampus or cerebellum. In contrast, we couldn’t detect PKH26-labled EVs in untreated mice, 24-h after administration.


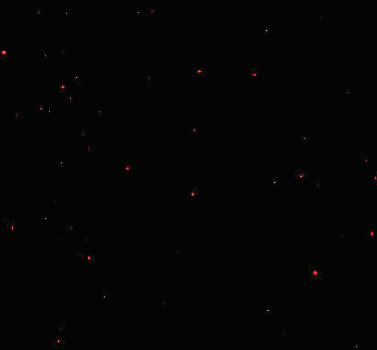

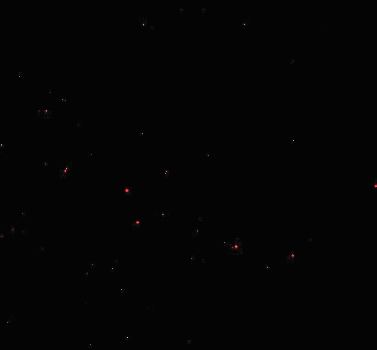

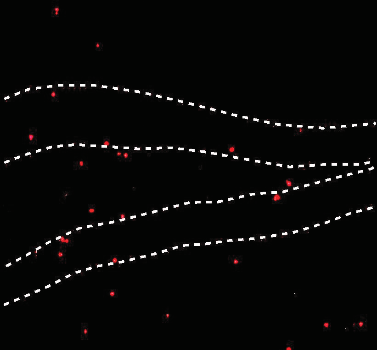

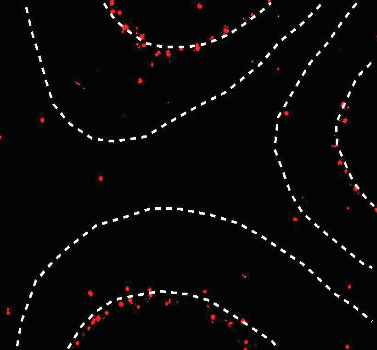

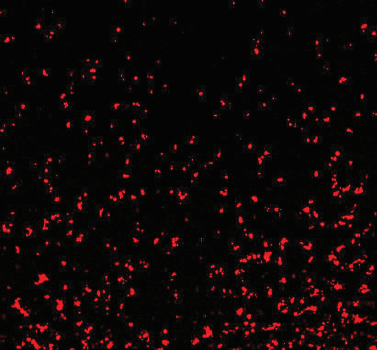

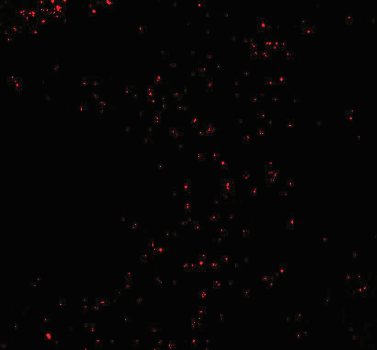

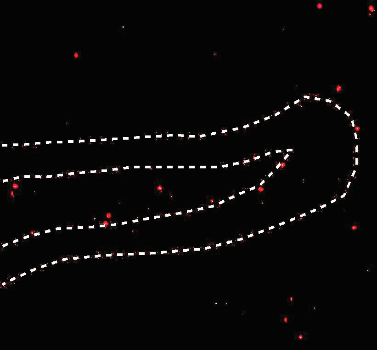

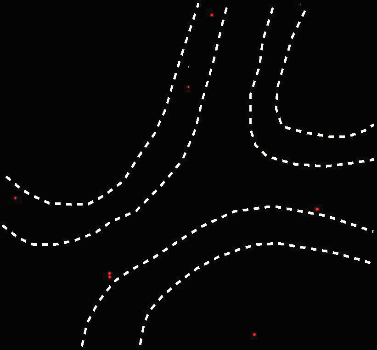

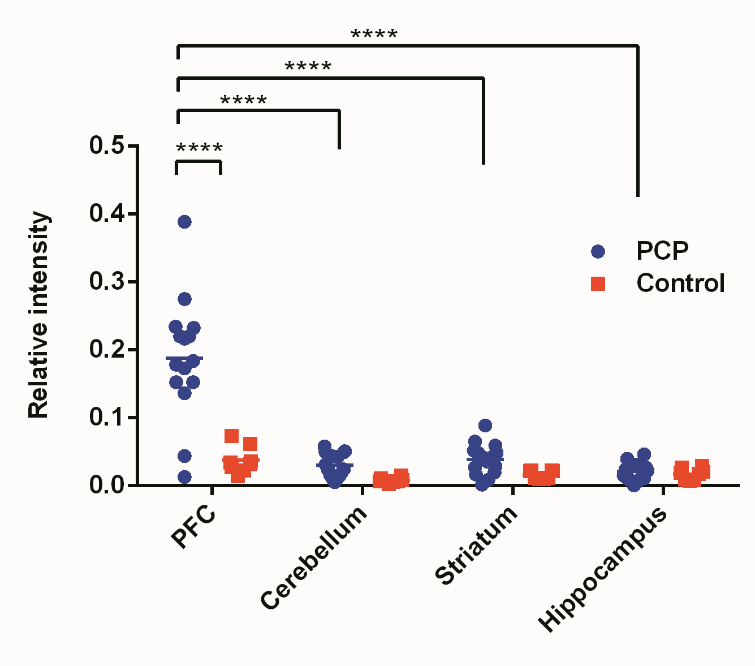

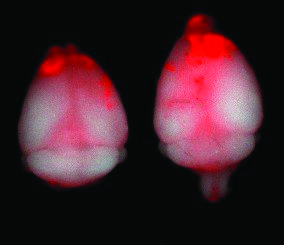

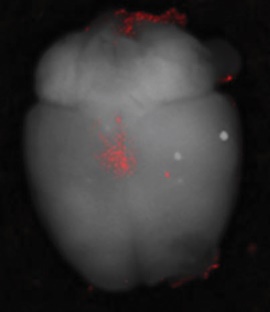

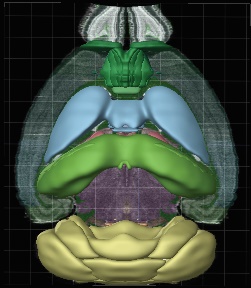


**PCP**

**Control**

**PFC**

**Striatum**

**Hippocampus**

**Cerebellum**

**PKH26**

100 µm

**PKH26**

100 µm

**PKH26**

100 µm

**PKH26**

100 µm

**PKH26**

100 µm

**PKH26**

100 µm

**PKH26**

100 µm

**PKH26**

100 µm

**B**

**C**

**A**

Supplementery Figure 3: Distribution of PKH-labeled EVs in the brain of PCP-treated mice and control mice. Mice treated with PCP and control mice (n=2) were intranasally administered with PKH26-labeled MSC-EVs and perfused 24h later. (A) Ex vivo fluorescent whole brain imaging of PKH-26-labeled MSC-EVs, showing aggregation of EVs in the frontal brain areas. (B) Representative images (cryostat sections) of PKH26-labeled EVs in different brain regions, indicating highest presence of MSC-EVs in the PFC. (C) Quantitative comparison of the EVs’ distribution. Results displayed as mean ± SEM. ****p<0.0001. Relevant brain sections were adapted from the Allen Mouse Brain 3D Atlas (dark green, prefrontal cortex; blue, striatum; green, hippocampus; yellow, cerebellum).

Statistical analysis:

For this test, to account for the experimental design with two factors (treatment and brain area), and unequal sample sizes in the treatment factor, we used linear mixed models with random mouse effects, followed by analysis of variance (ANOVA) and post-hoc analysis of difference in intensity between the control and PCP group, as well as intensity differences between the PFC and other brain areas among the PCP group. The analysis was performed using the R package lme4.
